# Leiomyoma: a case report of a rare benign tumor of the female urethra

**DOI:** 10.11604/pamj.2015.22.111.7785

**Published:** 2015-10-08

**Authors:** Amine Slaoui, Abdelouahed Lasri, Tarik Karmouni, Khalid Elkhader, Abdelatif Koutani, Ahmed Ibn Attaya

**Affiliations:** 1 Urology B, Ibn Sina, University Hospital, Rabat, Morocco

**Keywords:** Leiomyoma, smooth muscle, tumor, urethra

## Abstract

Leiomyoma is a benign smooth muscle tumor which is rarely found in urethra. It often appears in females during their reproductive age (from menarche to menopause); the mean age of their appearance is approximately 41 years. Less than 100 cases were reported in the literature. We hereby report a case of a 52-year-old White woman who presented with complaints of dysuria and urinary tract infection. The mass was completely removed by transvaginal excision with a rim of normal tissue. Histopathological studies confirmed the urethral leiomyoma. The patient remained asymptomatic and there was no evidence of recurrence in the followup.

## Introduction

Leiomyoma is a benign smooth muscle tumor which is rarely found in urethra. [1]. It often appear in females during their reproductive age (from menarche to menopause); the mean age of their appearance is approximately 41 years [2]. Less than 100 cases were reported in the literature [3].

## Patient and observation

**Figure 1:**
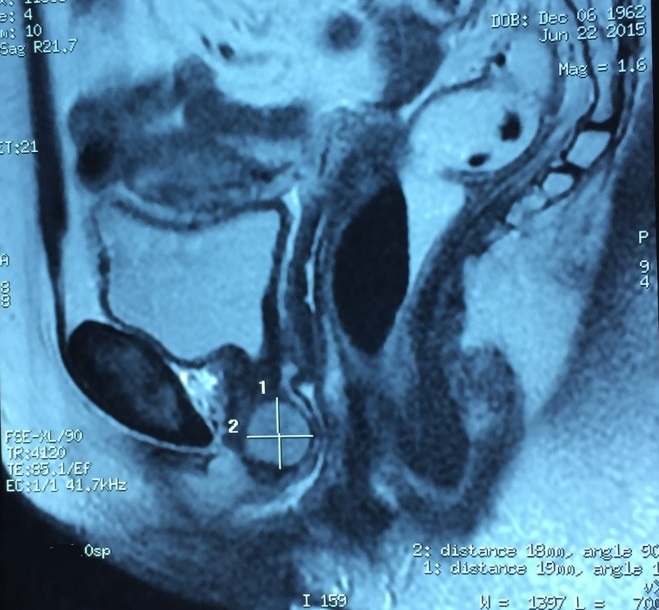
Sagittal section of a pelvic MRI showing a urethral leiomyoma

**Figure 2:**
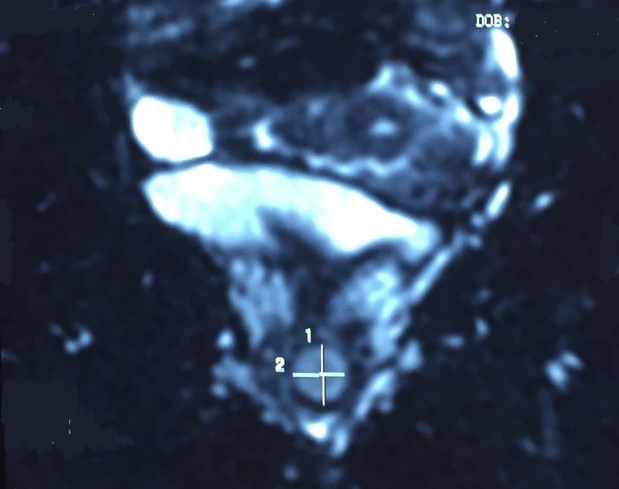
Coronal section of a pelvic MRI showing a urethral leiomyoma

**Figure 3:**
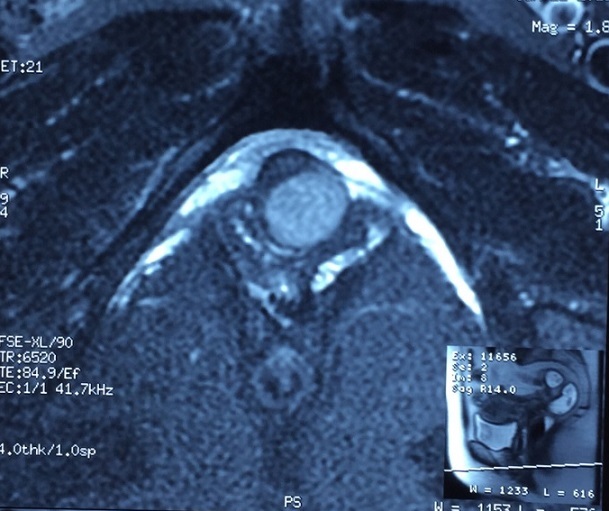
Cross section of a pelvic MRI showing a urethral leiomyoma

**Figure 4:**
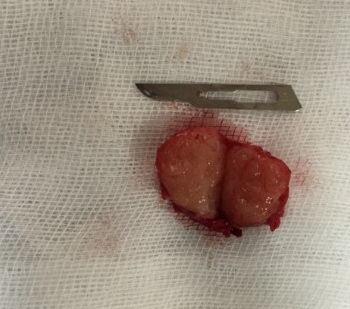
Surgical specimen after procedure

We hereby report a case of a 52-year-old White woman who presented with complaints of dysuria and urinary tract infection and feeling of nodulation in her vagina for the last 3 years. Her medical history revealed that she had no chronic disease. On physical examination, we detected a 2 × 2,5 cm painless hard tumor without signs of inflammation. Blood biochemistry and hemogram were within normal limits. Urinalysis was normal. The MRI revealed normal kidneys and large capacity bladder, with a mass located in the proximal third of the urethra and measuring 19x18 mm (Figure 1, Figure 2, Figure 3). The mass was completely removed by transvaginal excision with a rim of normal tissue (Figure 4). The urethral mucosa was intact. Patient was catheterized for 48 hours and voided well on catheter removal. Histopathological studies confirmed the urethral leiomyoma. The patient remained asymptomatic and there was no evidence of recurrence in the followup.

## Discussion

Buttner described the first case of leiomyoma on 1894. Although mostly observed in females of childbearing age, no age or gender is exempt [3]. Leiomyomas are benign tumors of the smooth muscles. They tend to be relatively common in the genitourinary and gastrointestinal tracts, the occurrences are less frequent in the skin and rare in the deep tissues. In general, soft tissue leiomyomas cause little morbidity. Leiomyomas can be classified under three categories: cutaneous leiomyoma (leiomyoma cutis), angiomyomas (vascular leiomyomas), and leiomyomas of deep soft tissue [1]. Urethral leiomyomas are, in fact, classified under leiomyomas of deep soft tissue and are rare benign mesenchymal tumors that originate from the smooth muscle of the urethra [4]. Leiomyoma of the urethra affects women more often than men [5]. It often appear in females during their reproductive age (from menarche to menopause); the mean age of their appearance is approximately 41 years [3]. Distal urethra can be affected but proximal segment is the most common site [6]. Urinary tract infection, a mass, dyspareunia, urinary retention, and irritative lower urinary tract symptoms are the most commonly reported symptoms. The tumor has been reported to enlarge during pregnancy and shrink after delivery, suggesting a possible hormonal dependence [7]. The differential diagnosis is represented by urethrocele, a urethral diverticulum, caruncle, and malignancy. To distinguish the differential diagnosis of urethral leiomyoma a careful clinical examination, urethroscopy, and radiological examination of the lower urinary tract are essential. However a pathological examination of the surgical pieces is mandatory not to disregard a neoplastic involvement. MRI may also help [8]. It is important to remember that no malignant transformation or recurrence have been reported. The treatment of choice is a local surgical resection [9].

## Conclusion

Leiomyoma is a benign smooth muscle tumor which is rarely found in urethra. It often appear in females during their reproductive age. A careful clinical examination, urethroscopy, and radiological examination of the lower urinary tract are essential. The treatment of choice is a local surgical resection. However a pathological examination of the surgical pieces is mandatory not to disregard a neoplastic involvement.

## References

[1] Rivière P, Bodin R, Bernard G, Deligne E, Peyromaure M, Ponties JE (2004). Leiomyoma of the female urethra. Prog Urol..

[2] Chong KM, Chuang j, Tsai YL, Hwang JL (2006). A rapidly growing paraurethral myoma with profuse bleeding from a mucosal vessel: report of a case. GynecolObstet Invest.

[3] Pahwa M, Saifee Y, Pahwa AR, Gupta M (2012). Leiomyoma of the female urethra-a rare tumor: case report and review of the literature. Case RepUrol..

[4] Dioszeghy F, Kiss A, Kondas J (1998). Leiomyoma of the female urethra. Int Urol Nephrol..

[5] Goldman HB, McAchran SE, MacLennan GT (2007). Leiomyoma of the urethra and bladder. J Urol..

[6] Lee MC, Lee SD, Kuo HT, Huang TW (1995). Obstructive leiomyoma of the female urethra: report of a case. J Urol..

[7] Fry M, Wheeler JS, Mata JA, Culkin DJ, St Martin E, Venable DD (1988). Leiomyoma of the female urethra. J Urol..

[8] Pavlica P, Bartolone A, Gaudiano C, Barozzi L (2004). Female paraurethral leiomyoma: ultrasonographic and magnetic resonance imaging findings. Acta Radiol..

[9] Deka PM, Rajeev TP (2003). Leiomyoma of the female urethra: a case report. Urol Int..

